# Clinical significance of hiatus hernia on Barrett’s oesophagus: a scoping review

**DOI:** 10.1007/s00423-026-03981-z

**Published:** 2026-02-19

**Authors:** Lee S. Kyang, Nurojan Vivekanandamoorthy, Manjunath Siddaiah-Subramanya

**Affiliations:** 1 Department of Upper Gastrointestinal Surgery, Bankstown-Lidcombe Hospital, New South Wales, Bankstown 2200 Australia; 2 School of Medicine, The University of New South Wales Australia, NSW Sydney, Australia; 3 School of Medicine, Western Sydney University, NSW Sydney, Australia

**Keywords:** Barrett’s oesophagus, Barrett’s esophagus, Hiatus hernia, Hiatal hernia, Risk factor, Cancer, Scoping review

## Abstract

**Background:**

Hiatal hernia (HH) is commonly observed in patients with Barrett’s oesophagus (BO), a premalignant condition that may progress to oesophageal adenocarcinoma (OAC). While HH has been implicated in BO pathogenesis, it is not formally recognised as a major risk factor in leading international clinical guidelines. This systematic scoping review aimed to evaluate the role of HH in the development, progression, and treatment outcomes of BO.

**Methods:**

A systematic scoping review was conducted, searching through databases (PubMed, Medline, Embase, Scopus).

**Results:**

A total of sixty-six articles were included with majority comprised of observational studies. HH was strongly associated with BO development, particularly in cases with larger hernia size (> 2–4 cm) and long-segment BO. While inconsistent, there could BO a trend towards dysplastic and malignant progression of BO in HH. HH may impair the efficacy of radiofrequency ablation, with larger hernias requiring more treatment sessions.

**Conclusion:**

Current evidence supports HH as a significant risk factor for BO onset. Its role in progression and management warrants further investigation. Surgical repair may be beneficial in selected asymptomatic patients, especially those with hernias ≥ 4 cm and established BO, to restore the gastro-oesophageal anatomy and eliminate reflux-prone micro-environment.

**Supplementary Information:**

The online version contains supplementary material available at 10.1007/s00423-026-03981-z.

## Background

Barrett’s oesophagus (BO) is defined as the metaplastic transformation of the normal squamous epithelial lining of the oesophagus into specialised intestinal-type columnar epithelium [1]. It arises as a consequence of chronic mucosal injury from gastro-oesophageal reflux disease (GORD)[2]. The true prevalence of BO is difficult to ascertain, as many patients are asymptomatic [3]. However, BO has been estimated to BO present in 1–2% of all patients receiving endoscopy for any indication and in approximately 5–15% in patients with chronic reflux symptoms [4].

BO is a clinically significant condition as it serves as a precursor to oesophageal adenocarcinoma. Compared to the general population, patients with BO have an estimated 10- to 55-fold increased risk compared to the general population [5]. The progression typically follows a metaplasia–dysplasia–adenocarcinoma sequence, with the presence of dysplasia markedly elevating the risk of malignant transformation [6]. Treatment options of BO with dysplasia include endoscopic mucosal resection (EMR), radiofrequency ablation (RFA) [7] and cryoablation [8]. Surgical intervention with fundoplication may also promote BO regression [9].

Diagnosis of BO generally requires two key components: (1) endoscopic evidence of columnar-lined epithelium (CLE) above the gastro-oesophageal junction (GOJ), anatomically demarcated by the proximal margin of the gastric rugal folds [10]; and (2) histopathological confirmation of intestinal metaplasia (IM), identified by the presence of goblet cells within biopsies obtained from the endoscopically abnormal segment [11]. Notably, guidelines vary slightly—most prominently, the British Society of Gastroenterology and the Asia-Pacific guidelines do not mandate the presence of IM for diagnosis [12].

Hiatal hernia (HH), defined as the proximal displacement of the stomach or other abdominal viscera through the diaphragmatic hiatus into the mediastinum [13], is a common endoscopic finding with an estimated prevalence exceeding 20% [14]. Although it has not yet been officially acknowledged as a significant risk factor in international guidelines [15–18], it has been proposed as a risk factor for the development of BO [19, 20]. The majority of BO patients have large HHs and advanced GORD [21]. HH often increases in size over time if left untreated [9]. It is strongly linked to heartburn and acid regurgitation when the size exceeds 2 cm [22]. This raises important clinical questions regarding the potential contribution of HH to BO pathogenesis. Specifically, even though surgical repair of HH is not usually advised for small asymptomatic sliding hernias [23], should HH alone justify surgical intervention in select cases to mitigate the risk of BO or its progression? Therefore, this systematic scoping review aimed to synthesise the evidence on the role of HH as a risk factor for BO. In addition, we examined the risk of dysplastic and malignant progression of BO in the presence of HH and explored how HH may influence BO management, with the overarching goal of informing patient counselling and guiding surgical decision-making.

## Methods

A scoping review was performed in accordance to the recommended methodology described by Arksey and O’Malley [24] and reported according to the Preferred Reporting Items for Systematic Reviews and Meta-analyses extension for Scoping Reviews (PRISMA-ScR) guidelines [25].

### Research questions

The review was conducted with an aim to evaluate the clinical significance of HH on BO. The research questions for this scoping review were:

1. Is there any association of HH with.

- Development of BO.
- Dysplastic progression of BO.
- Malignant progression of BO.

2. What is the size of HH that predisposes these risks?
3. How does presence of HH impact on the treatment of BO?

### Identification of relevant studies

An electronic search of the available literature was performed in the following databases from year 1979 to 2025: PubMed, Medline, Embase and Scopus. The search strategy consisted of the keywords and subject headings: “hiatus hernia”, “paraesophageal hernia”, “sliding hiatal hernia” and “Barrett oesophagus”.

### Study selection

To be selected for inclusion for the review, articles must either investigate the risk of HH on the development or progression of BO, or report on the implication of HH on the treatment of BO. Only cohort studies or randomised-controlled trials in the English language were eligible for inclusion. Exclusion criteria included patients under 18 years old, review articles, editorial and case reports.

**Fig. 1:**
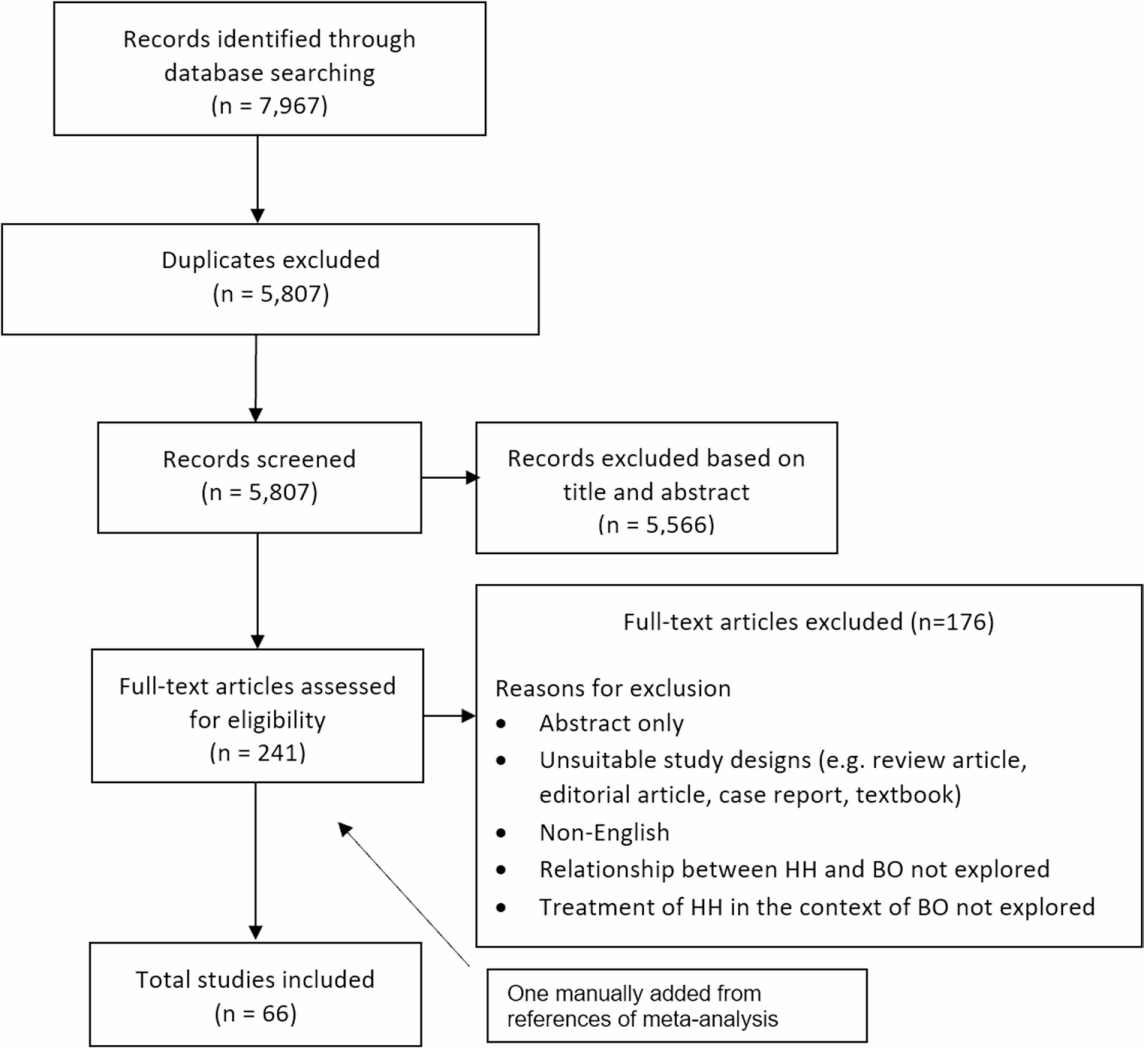
This data is mandatory please provide

The titles and abstracts of the initial search were screened by two authors (LSK and NV) independently. Eligible studies were then selected after independent evaluation of the full text. Disagreement on the study relevance was reviewed by discussion and consensus involving another independent author (MSS). In total, 66 articles were included with one being manually added after searching through the reference lists of relevant papers. Figure 1 provides an overview of the study selection process.

### Data summarisation

The eligible articles were retrieved and data were extracted into a standardised database in the form of Microsoft Excel. Information collected were: authors, years of publication, study location, study design, number of patients, study population, definition of BO and HH, aims of study, overview of methods, outcome measures and important results. Following the compilation of data, the results were organised into sections aligned with the research questions, addressing the role of HH in Barrett’s oesophagus: “risk of BO development”, “risk of dysplastic and malignant progression in BO”, and “impact of HH on BO treatment outcomes”.

## Results

The characteristics and overview of all included studies were summarised in Table 1. Majority of them were observational studies, comprising prospective cohort (*n* = 26), retrospective cohort (*n* = 19), case-control (*n* = 12), and cross-sectional (*n* = 8) designs. No randomised controlled trials were identified. Sample sizes ranged from 24 to 25,536 participants. Studies originated from 28 countries, with the United States contributing the largest proportion (*n* = 27). Non-U.S. studies included populations from Asia (*n* = 15, e.g., Japan, South Korea, China), Europe (*n* = 17, e.g., Italy, Germany, Turkey) and other regions (*n* = 8, e.g., Saudi Arabia, Chile, Iran). Publication years ranged from 1985 to 2023, with the majority (75%) conducted between 2005 and 2023.

**Table 1:** Characteristics of included studies

Authors	Years of publication	Location	Study design	Number of participants	Study population	Aims of study	Overview of methods	Outcome measures
Abrams et al. [26]	2008	USA	Retrospective cross-sectional	2100	Patients undergoing upper endoscopy (excluded prior BO/EAC)	Assess racial disparities in BO prevalence and risk factors (including HH)	Reviewed endoscopy records, excluded prior cases, multivariable logistic regression	BO prevalence by race, risk factors (sex, age, reflux, HH)
Akiyama et al. [27]	2012	USA	Retrospective cohort	45	BO patients undergoing RFA	Assess association of acid control/HH size with RFA outcomes • CE = endoscopically defined as 100% reduction in BO surface area following RFA	Retrospective review, pH monitoring, RFA procedures, multivariate analysis	Complete elimination (CE) of BO, reduction in BO surface area
Akiyama et al. [28]	2009	Japan	Retrospective cohort	869	Japanese patients undergoing upper endoscopy (374 BO cases, 47 with progression)	Determine prevalence/progression of BO and associated risk factors	Retrospective review of endoscopic records, logistic regression	Prevalence/progression rates, risk factors (ORs)
Alnasser et al. [29]	2019	Canda	Retrospective cohort	518	Patients with confirmed BO undergoing surveillance	Identify predictors of dysplastic/neoplastic progression in BO	Cox regression; generalized estimating equation analysis	Progression from nondysplastic BO to dysplasia/EAC
Alsahafi et al. [30]	2020	Saudi Arabia	Retrospective cohort	2,805	Adults undergoing EGD for any indication	Determine BO prevalence and risk factors (including HH)	Retrospective review of endoscopy database; multivariate regression analysis	BO prevalence, association with HH, demographics
Amano et al. [31]	2006	Japan	Prospective cohort study	1,668	Patients undergoing esophagogastroduodenoscopy for symptoms or check-ups	Investigate prevalence/risk factors for BO with intestinal predominant mucin phenotype	Targeted biopsies after crystal violet chromoendoscopy; immunohistochemical phenotyping	Association of HH with BO subtypes
Anandasabapathy et al. [32]	2007	USA	Retrospective cohort	109	Newly diagnosed BO patients	Identify factors predicting higher pathologic grades (HGD/EAC) in BO patients	Logistic regression analysis of clinical/endoscopic factors	Association of HH size with dysplasia severity
Asayama et al. [33]	2005	Japan	Retrospective cohort	100	Patients with SSBO (< 3 cm)	Assess SSBO elongation over time; identify predictors.	Endoscopic follow-up (mean 76.2 months), Kaplan-Meier analysis, Cox regression.	Cumulative probability of SSBO elongation; predictors (HH, esophagitis, initial SSBO length).
Asreah et. al. [34]	2021	Iraq	Cross-sectional observational	139	GORD patients with reflux symptoms referred for endoscopy	To study risk factors for erosive esophagitis (EE) and BO in GORD patients	Endoscopic evaluation, Los Angeles classification for EE, multivariate analysis for risk factors	Prevalence of BO/EE, association with HH
Avidan et al. [35]	2002	USA	Retrospective case-control	Cases: 131 with HGD/AC; Controls: 3,359 (2,170 non-GORD + 1,189 BO)	Patients with Barrett’s oesophagus, high-grade dysplasia (HGD), or esophageal adenocarcinoma (EAC)	Identify risk factors for progression from BO to HGD/EAC	Logistic regression; endoscopic/histologic evaluation; pH-metry and manometry	Association of HH size, BO length, acid reflux severity
Avidan et al. [35]	2002	USA	Case-control	485 (256 BO cases, 229 controls)	GORD patients (excluded prior surgery; BO confirmed histologically vs. nonerosive GORD controls)	Compare GORD patients with/without BO to identify risk factors (HH, reflux, smoking).	Structured symptom interviews, endoscopy, manometry, 24-hr pH monitoring. Multivariate logistic regression.	BO prevalence, association with HH, acid exposure, smoking, alcohol.
Balasubramanian et al. [36]	2012	USA	Prospective observational	1,058	GORD patients undergoing upper endoscopy	1. Determine CLE prevalence in GORD patients. 2. Identify predictors of CLE.	Prospective analysis of GORD patients. Univariate/multivariate logistic regression.	Prevalence of CLE, predictors (heartburn duration, race, HH).
Bani-Hani et al. [37]	2005	UK	Retrospective cohort	597	Patients with columnar-lined (Barrett’s) oesophagus	Identify risk factors for progression to esophageal adenocarcinoma in BO patients	Univariate and multivariate analysis of clinical/histologic factors	Risk factors (ORs) for adenocarcinoma progression
Brzacki et al. [38]	2018	Serbia	Prospective cohort	676	GORD patients with chronic reflux symptoms	Investigate BO prevalence and risk factors in GORD patients	Endoscopy with biopsy; statistical analysis of HH and symptoms	BO prevalence, risk factors (age, sex, HH)
Cameron AJ [39]	1999	USA	Prospective observational	167 (46 BO, 18 SSBO, 103 controls)	Patients referred for upper GI endoscopy for clinical indications and BO	(1) Determine HH prevalence/size in BO vs. controls. (2) Link HH to BO pathogenesis.	Endoscopic measurement of HH length/hiatus width. Excluded prior GI evaluations. Statistical comparisons (χ², t-tests).	HH prevalence, hernia size, hiatus width.
Campos et al. [40]	2001	USA	Case-comparison	502 GORD patients	GORD patients with/without BO (67 short-segment BO, 107 long-segment BO)	Identify predictive factors for BO presence/extent in GORD patients	Multivariate analysis of clinical, endoscopic, and pH/bilirubin monitoring data	Predictive factors for BO, HH size impact
Chen et al. [41]	2019	Taiwan	Retrospective cohort	3,385	General population undergoing health check-ups	Determine BO prevalence and risk factors in Taiwan	Endoscopy with biopsy; multivariate analysis of risk factors	BO prevalence, risk factors (age, sex, HH, tea)
Coleman et al. [42]	2014	Ireland	Retrospective population-based cohort study	3,148	BO patients with specialized intestinal metaplasia	Investigate symptoms/endoscopic features and progression risk	Hospital record review, linkage to cancer registry, Cox models	Progression to HGD/EAC
Conio et al. [43]	2002	Italy (multi-centre)	Case-control	600 (149 BO, 143 E, 308 C)	BO patients, esophagitis patients, hospital controls (non-GI conditions)	Identify risk factors for BO and esophagitis (including HH)	Questionnaires, endoscopy, histology, logistic regression	Association of HH, GORD symptoms, alcohol, and smoking with BO/esophagitis
Csendes et al. [44]	2002	Chile	Prospective cohort	582 (408 BO, 174 IMC)	LSBO (> 3 cm), SSBO (< 3 cm), IMC patients.	Compare clinical/functional features of LSBO, SSBO, IMC.	Endoscopy, manometry, 24-h pH/bilirubin monitoring. Statistical comparisons (χ², t-tests).	GORD symptoms, HH prevalence, acid/bile reflux, dysplasia.
Dhawan et al. [45]	2001	India	Cross-sectional observational study	271	Patients undergoing diagnostic upper GI endoscopy for dyspepsia/heartburn	To study prevalence and associations of short-segment SCE in distal oesophagus among Indians	Endoscopy, biopsies, histopathology (H&E and alcian blue/PAS), statistical analysis (χ², t-test)	Prevalence of SCE, associations with GORD, HH
Dickman et al. [46]	2005	USA (multi-center)	Retrospective cohort analysis	263	BO patients (LSBO/SSBO) from GI clinics/labs	Identify clinical factors correlating with BO length.	Questionnaires, endoscopy, histology. Regression and t-tests for analysis.	BO length correlation with HH size, smoking, medications (PPI/H2RA), dysplasia.
Dina et al. [47]	2015	Romania	Prospective cohort	1,261	Hospitalised patients referred for endoscopy (excluded upper GI bleeding, tumors)	Investigate BO prevalence and risk factors (GORD, HP, HH, BMI, smoking, alcohol)	Clinical/demographic data collection, endoscopy, HP testing via urease, biopsies. Statistical analysis with SPSS.	BO prevalence, associations with GORD, HP, HH, BMI, smoking, alcohol
Gashi et al. [48]	2013	Albania	Retrospective cohort study	58	Patients with endoscopically and histologically confirmed BO	To correlate histopathology with clinical/endoscopic findings and identify BO risk factors	Endoscopy, biopsies, histopathology, statistical analysis (χ²-test)	HH prevalence, BO segment length, H. pylori status
Gashi et al. [49]	2011	Republic of Kosova	Prospective cohort	42	GORD patients with BO	Identify predictive factors for BO in GORD patients	Endoscopy, biopsies, H. pylori testing, analysis of demographics and risk factors	Association of HH, obesity, H. pylori, and demographics with BO
Iwaya et al. [50]	2019	Canada	Retrospective cohort	324 (145 LSBO, 179 SSBO)	EAC patients with LSBO or SSBO undergoing EMR.	Identify factors that are strongly associated with development of EAC arising from LSBO vs. SSBO.	Analysis of a prospective database. Logistic regression for variables (smoking, statins, HH).	HH prevalence, smoking pack-years, statin use, GORD symptoms.
Jonaitis et al. [51]	2011	Lithuania	Prospective cross-sectional	4032 (193 analyzed)	Patients referred for upper endoscopy with upper GI/alarm symptoms	Determine prevalence and risk factors of BO in a high H. pylori prevalence area	Endoscopy, biopsies, H. pylori testing, logistic regression	Prevalence of BO, risk factors (HH size, H. pylori, smoking)
Kim et al. [52]	2005	South Korea (multi-centre)	Prospective cohort	992 patients	Patients undergoing first diagnostic endoscopy due to various indications	Assess BO prevalence in Korea.	Endoscopy with biopsies, histology (Alcian blue).	BO prevalence, association with reflux esophagitis/HH.
Korst et al. [53]	2011	USA	Phase II clinical trial	67	BO patients undergoing radiofrequency ablation (RFA)	Evaluate HH size and BO length impact on RFA success	RFA treatment, endoscopic measurements, statistical analysis	RFA success rate, sessions required, nonhealing rates (endoscopically replacement of the ablated area with an eschar with no regeneration of mucosa 2 months later)
Krishnamoorthi et al. [54]	2016	UK	Population-based cohort	9,660	BO patients from primary care records.	Identify predictors of progression to esophageal carcinoma (EC) in BO patients.	Cox proportional hazards models; time-varying marginal structural models for medications.	Incidence rates, hazard ratios (HRs) for progression.
Krishnan et al. [55]	2012	USA	Retrospective cohort	37	BO patients undergoing RFA	Assess impact of reflux exposure on persistent IM after RFA	High-resolution manometry, 24-h impedance-pH monitoring, RFA protocol, biopsies	Complete response (CR) vs. incomplete response (ICR) based on IM eradication CR = complete eradication of all dysplasia and IM at third endoscopy ICR = residual dysplasia or IM at third endoscopy
Kubota et al. [56]	2022	Japan	Prospective cohort	10,122	Japanese individuals undergoing routine esophagogastroduodenoscopy (EGD) during health checkups (excluded gastric surgery, poor EGD images, missing data)	1. Assess BO risk factors (H. pylori status, hiatal hernia, PPI use, etc.). 2. Compare BO prevalence in H. pylori-negative, H. pylori-positive, and post-eradication groups.	Retrospective analysis of EGD images and questionnaires; H. pylori status via serum IgG; multivariate logistic regression; subgroup analysis.	BO prevalence, LSBO/SSBO rates; odds ratios (ORs) for risk factors.
Kuo et al. [57]	2010	Taiwan	Prospective cohort	736	GORD patients undergoing endoscopy (excluded prior gastric surgery, esophageal varices)	Determine BO frequency and risk factors in Taiwanese GORD patients.	Structured symptom questionnaires, standardized endoscopy (Prague C&M), histopathology. Logistic regression analysis.	BO prevalence, risk factors (HH, GORD duration), dysplasia progression.
Kwon et al. [58]	2020	USA	Case-control study	118,750 BO patients	Patients in the Nationwide Inpatient Sample (NIS) database with BO diagnostic codes (24,030 with HH; 94,720 without HH)	Determine HH association with BO, dysplasia, and ablation outcomes.	NIS 2016 database analysis; propensity score matching; multivariate logistic regression.	Adjusted odds ratios (aOR) for BO, dysplasia (low/high grade), and ablation rates.
Lee et al. [59]	2010	South Korea (multi-centre)	Cross-sectional prospective	2,048	Patients with GI symptoms undergoing endoscopy	Assess BO prevalence and risk factors in Koreans	Endoscopy with biopsies; validated symptom questionnaire; histologic confirmation of SIM	Prevalence of BO and association with HH
Lord et al. [21]	2009	USA (multi-center)	Retrospective cohort study	160 patients	GORD patients (NERD, mild ERD, severe ERD, BO)	Investigate mechanical factors (HH, LES) in GORD spectrum and Nissen fundoplication outcomes.	Preoperative endoscopy, manometry, pH monitoring; postoperative symptom assessment.	Prevalence of HH, LES pressures, acid exposure, postoperative symptom control.
Mathew et al. [60]	2011	India	Prospective cohort	278 patients	Patients with GORD	Determine BO prevalence and risk factors (HH, age, symptoms).	Endoscopy, symptom questionnaires, biopsies.	BO prevalence, risk factors (ORs for HH, age, eructation).
Mohamed Hussein et al. [61]	2021	UK	Retrospective cohort study	147	Patients with Barrett’s oesophagus and LGD	Compare progression risk in confirmed vs. downstaged LGD; assess risk factors	Retrospective analysis of tertiary referrals, biopsies reviewed by experts, Cox regression	Progression to HGD/EAC
Nguyen et al. [62]	2014	USA	Cross-sectional case-control	1952 (301 BO cases)	Veterans (mostly male, African American [AA] and non-Hispanic White [NHW])	Compare BO prevalence and risk factors between AAs and NHWs	Endoscopy, questionnaires, logistic regression	BO prevalence by race, HH size, H. pylori, WHR
Öberg et al. [63]	1998	USA	Prospective observational	262	Patients with foregut symptoms (excluded prior surgery/motility disorders).	Compare acid/bile exposure and HH in SSBO vs. LSBO, erosive esophagitis, and controls.	Endoscopy, manometry, 24-hr pH/bilirubin monitoring. Statistical analysis (Kruskal-Wallis, Mann-Whitney).	Acid/bile exposure, HH prevalence, LES function, BO length.
Ödemiş et al.[64]	2009	Turkey	Prospective cohort	1,000	Patients undergoing endoscopy for any indication (excluded prior BO, GI bleeding)	Assess prevalence of BO and association with GOJ integrity (normal, widened, HH)	Structured symptom interviews, standardized endoscopy (GOJ classified into 3 types), histopathology. Statistical analysis with SPSS.	Prevalence of BO, association with GOJ integrity, reflux symptoms, erosive esophagitis
Park et al. [65]	2009	South Korea (multi-centre)	Prospective cohort study	25,536	Patients undergoing routine health check-ups with upper endoscopy screening	To evaluate BO prevalence and identify risk factors in the Korean population	Endoscopy, biopsies, histopathology, statistical analysis (multivariate logistic regression)	BO prevalence, risk factors (ORs)
Peng et al. [66]	2009	China	Cross-sectional study	2,580	Asymptomatic adults undergoing routine upper endoscopy	Determine prevalence and risk factors of silent GORD (erosive esophagitis/BO in asymptomatic individuals)	Endoscopy with biopsies; validated symptom questionnaire; histologic confirmation of SIM	Association of HH with BO in asymptomatic patients
Pohl et al. [67]	2013	Germany	Case-control	563 (113 no GORD, 188 GORD, 162 BO, 100 cancer/HGD)	Patients categorized into no GORD, GORD, BO, and cancer/HGD	Identify risk factors at different stages (GORD → BO → cancer)	Questionnaires, endoscopy, logistic regression	ORs for risk factors at each disease stage
Quach et al. [68]	2020	Vietnam	Cross-sectional	1,947	Vietnamese patients with upper GI symptoms	Assess BO prevalence, clinical characteristics, and risk factors in Vietnam	Endoscopy with biopsies using Prague C&M criteria; multivariate analysis	BO prevalence, risk factors (HH, reflux symptoms)
Rajendra et al. [69]	2004	Malaysia	Prospective cohort	1,985	Multiethnic patients (Malay, Chinese, Indian) undergoing endoscopy for upper GI symptoms (excluded peptic ulcers, prior surgery).	Assess ethnic differences in esophagitis and BO prevalence.	Endoscopy (Savary-Miller grading), biopsies, H. pylori testing. Statistical analysis via EpiInfo.	Prevalence of esophagitis/BO, association with ethnicity, HH, reflux symptoms.
Ringhofer et al. [70]	2008	Austria	Prospective cohort	102 patients	GORD patients with heartburn/regurgitation	Compare endoscopic vs. histopathologic findings at GOJ; assess HH impact on BO.	Multilevel biopsies (proximal/distal to rugal folds), histopathology (Pauli-Chandrasoma classification).	CLE/IM prevalence, HH association, biopsy yield.
Ronkainen et al. [71]	2005	Sweden	Population-based endoscopic study	1,000	Random sample of general adult population	Determine BO prevalence and risk factors in the general population	Endoscopy with biopsies; validated symptom questionnaire; histologic confirmation of SIM	Prevalence of BO and association with HH
Sarr et al. [72]	1985	USA	Retrospective cohort	1020 (84 with BO)	GORD patients undergoing esophagoscopy/biopsy	(1) Determine BO prevalence in GORD patients. (2) Compare BO vs. non-BO groups. (3) Compare BO with/without adenocarcinoma.	Review of pathology records (1975–1981). Excluded duplicates, squamous/gastric cancers, and infections. Chi-square analysis for comparisons.	BO prevalence, HH/stricture/ulcer associations, adenocarcinoma rates in BO.
Schlottmann et al. [73]	2018	USA	Retrospective review of prospective database	175	GORD patients diagnosed by 24-hour pH monitoring	To assess impact of HH presence/size on GORD presentation, esophageal function, reflux, mucosal injury	Symptom evaluation, barium swallow, high-resolution manometry, pH monitoring, endoscopy	LES pressure, DeMeester score, esophagitis, BO
Sgouros et al. [74]	2007	Greece	Prospective observational	619 patients, 258 controls	Patients with NERD, reflux esophagitis, Barrett’s oesophagus; controls with peptic ulcers	Investigate HH prevalence/axial length in NERD vs. erosive GORD and controls	Endoscopy with HH size measurement; statistical analysis of prevalence and axial length	HH prevalence, axial length (> 3 cm) association with GORD severity
Sharifi et al. [75]	2014	Iran	Prospective cohort	736	Iranian patients with GORD symptoms	Determine prevalence and risk factors for EE and BO	Upper endoscopy, biopsies, H. pylori testing, multivariate analysis	BO prevalence, association with HH, H. pylori, age
Sikkema et. al. [76]	2011	Netherlands (multi-centre)	Prospective cohort	713	BO ≥ 2 cm with ND/LGD; excluded prior HGD/EAC	Identify predictors of neoplastic progression (HGD/EAC) in BO	Endoscopic HH assessment; multivariable log-linear regression	Development of HGD/EAC over 4 years
Solanky et al. [77]	2019	USA (multi-centre)	Prospective cohort	619	NDBO and LGD patients	Identify predictors of progression from NDBO/LGD to HGD/EAC	Cox proportional hazards model; multivariate analysis	Impact of HH length on progression
Soroush et al. [78]	2019	USA	Retrospective cohort study	290	BO patients undergoing endoscopic eradication	Assess if time to CE-IM affects outcomes; evaluate HH impact on recurrence	Retrospective analysis (2006–2017); Kaplan-Meier/Cox models; CE-IM defined by endoscopy Main analysis: CE-IM defined as first post-EET endoscopy without endoscopic evidence of BO or IM Seconary analysis: CE-IM defined as two consecutive endoscopies after EET without endoscopic evidence of BO or IM	Recurrence of IM and dysplasia/EAC
Thiruvengadam et al. [79]	2020	USA	Retrospective cohort	460 BO patients	Patients with biopsy-documented BO	Identify risk factors for progression to HGD/EAC in BO patients.	Multivariate Cox regression with LASSO penalization.	Progression to HGD or EAC.
Toruner et al. [80]	2004	Turkey	Prospective cohort	395 patients	Dyspeptic patients undergoing gastroscopy	Determine BO prevalence and relationship with dyspeptic symptoms/HH.	Endoscopy, symptom questionnaires, biopsies (antrum, SCJ).	Prevalence of BO, association with HH, symptoms.
Tsoi et al. [7]	2020	Australia	Prospective Observational Study	152 patients	Dysplastic BO patients treated with RFA	Identify factors predicting poor RFA response, including HH characteristics	Divided patients into good/poor responders; compared HH presence, size, BO length, reflux esophagitis GR: eradication of dysplasia and IM within 3 or less treatment PR: patients requiring 4 or more treatment sessions	Complete eradication of dysplasia and intestinal metaplasia
Wakelin et al. [81]	2003	USA	Prospective cohort	24	Patients with BO diagnosed endoscopically (excluded dysplasia/cancer)	(1) Determine correlation between HH length and BO length. (2) Develop a predictive model for BO length using HH length and duration of esophageal acid exposure.	Endoscopy with biopsies for BO confirmation; 24-hour pH monitoring for acid exposure (% time pH < 4); HH length measured endoscopically; Pearson correlation and multiple linear regression.	Barrett’s oesophagus length; Hiatal hernia length; Duration of esophageal acid exposure (% time pH < 4).
Wang et al. [82]	2008	USA	Retrospective review	2,511	Patients undergoing upper endoscopy	Assess accuracy of endoscopy for BO diagnosis; identify predictors	Review of endoscopy and pathology reports from multiple centers	Confirmation rate of BO, predictors (HH, race, age, segment length)
Weiss et al. [83]	2023	France	Retrospective study	96	Patients with dysplastic BO treated with RFA	Identify predictive factors of RFA failure in dysplastic BO	Retrospective analysis (2011–2020 database); univariate/multivariate Cox models	RFA failure (persistence of IM or neoplastic progression)
Weston et al. [84]	2004	USA	Prospective cohort study	550 (324 in surveillance)	Veterans with newly diagnosed Barrett’s oesophagus	To identify endoscopic/histologic features predictive of Barrett’s dysplasia and cancer progression	Endoscopic surveillance with biopsy protocol, Cox regression for progression risk	Progression to HGD, intramucosal cancer, or invasive cancer
Weston et al. [85]	1999	USA	Prospective cohort study	99	Male veterans with BO undergoing long-term medical management	Identify predictors of complete regression of BO during medical therapy.	Endoscopy with biopsies, Lugol’s iodine chromoendoscopy, multivariate logistic regression.	Complete regression of BO (endoscopic + histologic)
Wu et al. [86]	2003	USA	Population-based case-control	1356 controls + 942 cases	Patients with esophageal (*n* = 222), gastric cardia (*n* = 277), and distal gastric (*n* = 443) adenocarcinomas	Evaluate associations between hiatal hernia, reflux conditions (including Barrett’s oesophagus), and adenocarcinoma	Unconditional logistic regression adjusted for demographics, smoking, BMI	Odds ratios (ORs) for cancer risk related to HH, reflux symptoms, and BMI
Xiong et. al. [87]	2010	China	Prospective cohort	2022	Patients undergoing upper endoscopy for UGI symptoms	Investigate the prevalence of BO and its risk factors	Endoscopy, biopsies, multivariate analysis	Confirmation rate of BO, predictors (HH, gender, age, reflux oesophagitis, BMI, heartburn)
Yilmaz et. al. [88]	2006	Turkey	Retrospective cohort	18,766	Patients undergoing upper endoscopy	Determine BO prevalence, erosive esophagitis, and concordance of endoscopic vs. histologic BO	Retrospective review of endoscopy records; biopsies from columnar epithelium	BO prevalence, HH presence, esophagitis rates

Definitions of BO and HH varied across studies. In most studies (*n* = 55), histopathological confirmation of IM was required for the diagnosis of BO. HH, on the other hand, was diagnosed endoscopically based on a separation of ≥ 2 cm between the GOJ and the diaphragmatic hiatus, as defined in 18 studies. One study used a threshold of ≥ 3 cm, while another used ≥ 1 cm. The remaining studies did not specify the size criteria used for the diagnosis of HH.

### Risk of BO development

A total of 46 studies reported on the association of HH with BO (Table 2). Of these, 41 studies demonstrated a strong positive association between HH and BO[21, 26, 31–36, 38–41, 43, 44, 46, 48, 49, 51, 52, 56–60, 62–75, 80, 81], with one study reporting an adjusted odds ratio (OR) of 10.9 [58] - indicating nearly an eleven-fold increased risk of developing BO in the presence of HH. Conversely, the absence of HH has been associated with complete regression of BO in some cases [85].

**Table 2:** Association of hiatus hernia with barrett’s oesophagus

Authors	Definition of BO	Definition of HH	Important results	Notes
Abrams et al. (2008) [26]	Confirmed by pathology (intestinal metaplasia); LSBO ≥ 3 cm	Presence noted during endoscopy; size not recorded	HH significantly associated with increased BO risk (OR 3.53).	HH size not recorded. Possible underdiagnosis in minorities.
Akiyama et al. (2009) [28]	Macroscopic endoscopic identification of abnormal columnar esophageal epithelium suggestive of columnar-lined distal oesophagus.	Distance between GOJ and diaphragmatic hiatus ≥ 2 cm	HH was significantly associated with progression of Barrett’s epithelium in univariate analysis (OR 1.99) but not multivariate.	HH ≥ 2 cm common in BO patients but not an independent risk factor for progression.
Alsahafi et al. (2020) [30]	Endoscopic: Salmon-colored mucosa ≥ 1 cm proximal to GOJ; Histologic: Intestinal metaplasia confirmation	Endoscopic observation of discordance between diaphragmatic impingement and GOJ	1. BO prevalence: 0.64% (endoscopic), 0.32% (histologic). 2. HH not associated with BO (OR 0.98, *p* = 0.97) on multivariate analysis.	HH not a significant factor. No data on dysplasia progression, HH size, or treatment impact.
Amano et al. (2006) [31]	Endoscopic columnar-appearing mucosa ≥ 5 mm above GOJ + histologic confirmation (goblet cells with acid mucin)	Presence of HH diagnosed endoscopically	1. 19.9% had BO 2. HH was an independent risk factor for BO with intestinal phenotype (OR 2.29, 95% CI 1.88–2.89, *p* < 0.001). 3. Dysplastic lesions were more common in intestinal-phenotype BO (15.1% vs. 0.9% in gastric phenotype, *p* < 0.001).	Focused on mucin phenotypes; included SSBO and LSBO.
Anandasabapathy et al. (2007) [32]	Endoscopic identification of squamocolumnar junction proximal to gastroesophageal junction + goblet cells	Endoscopic identification (not explicitly defined)	1. Univariate analysis: hiatal hernia size ≥ 4 cm: OR 10.63 (*P* = 0.014) was predictor of HGD/EAC. 2. Bivariate analysis: male and large hiatal hernia size (OR: 4.64, P 0.0049 and OR: 12.18, P 0.0197) were each independently associated with high grades of dysplasia when analysed in a pair	HH size ≥ 4 cm is a significant predictor. No direct recommendations for HH management in BO treatment provided.
Asayama et al. (2005) [33]	SSBO defined as columnar-lined oesophagus < 3 cm (Japanese Society for Esophageal Diseases criteria).	Hiatal hernia: Patent hiatus ≥ 2x width (retroflexed endoscopy).	1. 30% of SSBO elongated to ≥ 3 cm. 2. Predictors: HH (HR = 4.17, *p* < 0.05), severe reflux esophagitis (HR = 9.09, *p* = 0.01), initial SSBO ≥ 1 cm (HR = 3.93, *p* = 0.01).	
Asreah et al. (2021) [34]	Columnar epithelium in distal oesophagus confirmed by histopathology (intestinal metaplasia)	Endoscopically diagnosed hiatal hernia	HH significantly associated with BO (OR 17.2, *p* = 0.004)	HH size not specified; focuses on presence/absence.
Avidan et al. (2002) [35]	SIM with goblet cells in columnar-lined oesophagus (any length). Endoscopic irregular squamocolumnar junction.	HH: Gastric folds ≥ 2 cm above diaphragmatic hiatus.	1. HH prevalence: 76% in BO vs. 36% in controls (*P* < 0.001). 2. HH (*P* < 0.001) and frequent reflux episodes (*P* < 0.001) were top predictors of BO. 3. HH size was an independent risk factor for length of BO	Limitations: Male-dominated VA cohort, no dysplasia/malignancy data. Strengths: Standardized endoscopic criteria. Confounders: Alcohol/smoking interactions.
Balasubramanian et al. (2012) [36]	Columnar-lined oesophagus (CLE) with/without intestinal metaplasia.	Presence and size of hiatal hernia (no explicit size criteria).	1. CLE prevalence: 23.3%. 2. HH (OR 2.07, 95% CI:1.50–2.87,*P* < 0.01) was an independent RF for BO	
Brzacki et al. (2018) [38]	Columnar-lined oesophagus (CLE) ≥ 1 cm with IM confirmed by biopsy	Presence noted (no specific size criteria)	1. BO prevalence: 2.22%. 2. HH present in 80% of BO patients (*p* < 0.05).	
Cameron AJ (1999) [39]	Histological confirmation of intestinal metaplasia. SSBO = < 3 cm columnar epithelium with IM; LSBO = ≥ 3 cm	≥ 2 cm separation between GOJ and crural impression endoscopically; hiatus width estimated.	1. 96% of BO patients had HH ≥ 2 cm vs. 42% controls (*p* < 0.001). 2. HH was found in 72% of patients with SSBO, higher than controls (*p* < 0.05) 3. HH length: 3.95 cm (BO) vs. 2.77 cm (controls w/ oesophagitis; *p* = 0.003) vs. 2.86 cm (controls w/o oesophagitis (*p* = 0.005). 4. Hiatus width wider in BO (3.52 cm) vs. controls w/ oesophagitis (2.27 cm) and controls w/o oesophagitis (2.21 cm).	No patient in the control group was found to have LSBO
Campos et al. (2001) [40]	Columnar epithelium with intestinal metaplasia; short-segment (< 3 cm), long-segment (≥ 3 cm)	≥ 2 cm separation between GOJ and crural impression; size categorized (2–4 cm, > 4 cm)	1. HH > 4 cm: Strongest predictor of long-segment BO (OR 17.8). 2. HH > 4 cm (OR4.1) was independent RF for BO	Focuses on BO presence/extent, not dysplasia/malignancy.
Chen et al. (2019) [41]	Columnar epithelium ≥ 1 cm with intestinal metaplasia (IM) confirmed by biopsy	Endoscopic diagnosis (no specific size criteria)	1. BO prevalence: 2.6%. 2. HH (OR = 3.037) was significant RF for predicting BO 3. No dysplasia/cancer reported.	Focuses on general population; no dysplasia analysis.
Conio et al. (2002) [43]	Endoscopic displacement of squamocolumnar junction + intestinal metaplasia	Presence noted during endoscopy; size not measured	1. HH significantly associated with BO (OR 3.9, 95% CI 2.5-6.0) 2. Longer GORD symptoms increased BO risk.	HH size not analyzed. Controls may have recall bias.
Csendes et al. (2002) [44]	LSBO: >3 cm columnar epithelium with intestinal metaplasia (presence of goblet cells); SSBO: <3 cm.	Presence of HH recorded endoscopically (no explicit size criteria).	1. Patients with IMC had significantly lower frequencies of hiatal hernia (2.8%) vs. SSBO (30%) vs. LSBO (70.2%) 2. Between patients with SSBO and LSBO, significant differences were found in the frequency of hiatal hernia (both greater in the patients with LSBO).	
Dhawan et al. (2001) [45]	Specialized columnar epithelium (SCE) with goblet cells	> 2 cm distance between gastric folds and diaphragmatic hiatus	1. SCE prevalence: 6% (16/271). 2. SCE associated with histologic esophagitis (*p* < 0.001) but not HH (p = NS).	Focuses on Indian population; HH not linked to SCE.
Dickman et al. (2005) [46]	LSBO (≥ 3 cm), SSBO (< 3 cm); intestinal metaplasia confirmed by biopsy.	Displacement of GOJ ≥ 2 cm proximal to the diaphragmatic hiatus.	1. Longer HH correlated with longer BO (*r* = 0.22, *p* < 0.01). 2. A higher grade of dysplasia was correlated with a longer BO length (*r* = 0.124,*p* < 0.05) 3. Ors for having LSBO and SSBO are not significant (1.9; 95%CI 1-3.4)	
Dina et al. (2015) [47]	Endoscopic reddish mucosal areas + histologic intestinal metaplasia (no dysplasia). Short BO (< 3 cm), long BO (> 3 cm)	HH presence assessed via endoscopy (no size criteria provided)	1. BO prevalence: 2.85%. 2. GORD (*P* < 0.001) and HP (*P* = 0.02) linked to BO. 3. No association between HH and BO (22.9% in BO vs. 20.3% in non-BO; *P* = 0.7)	Limitations: No HH size criteria; potential misclassification of BO islands. Confounders: High cirrhosis rates in non-BO group. Strengths: Large sample size
Gashi et al. (2011) [49]	Endoscopic + histologic confirmation of intestinal metaplasia	Presence of hiatal hernia diagnosed endoscopically.	1. 64% of BO patients had HH. 2. HH more common in LSBO (66%) vs. SSBO (34%) (*p* < 0.05) 3.Low H. pylori prevalence (12%). 4. Male, age 50–59, smoking, obesity linked.	HH prevalent in BO, especially LSBO. Low H. pylori suggests protective role. Small sample size.
Gashi et al. (2013) [48]	Intestinal metaplasia confirmed by histopathology	HH presence assessed via endoscopy (no size criteria provided)	1. HH present in 69% (40/58) of BO patients (*p* < 0.01) 2. HH more common in long-segment BO (100% vs. 48.6% in short-segment, *p* < 0.001).	Emphasizes histopathology as gold standard; low H. pylori in BO suggests protective role.
Jonaitis et al. (2011) [51]	Intestinal metaplasia with goblet cells (biopsy-confirmed)	Measured in cm; >2 cm considered significant	HH > 2 cm strongly associated with BO (OR 5.22, 95%CI 1.86–14.64, *p* = 0.002).	HH size measured; Lithuanian rural population with high H. pylori.
Kim et al. (2005) [52]	Specialized columnar epithelium with goblet cells	HH ≥ 2 cm (distance between diaphragm and GOJ).	1. BO prevalence: 3.6% (LSBO 0.1%, SSBO 3.5%). 2. HH more common in BO group (22.2% vs. 8.9%, *p* = 0.02).	Lower BO prevalence than Western countries; adenocarcinoma rare in Korea.
Kubota et al. (2022) [56]	Endoscopic finding of ≥ 1 cm continuous salmon pink mucosa from GOJ (Asian-Pacific consensus). LSBO ≥ 3 cm; SSBO < 3 cm. No pathologic diagnosis	Hiatal hernia (HH) diagnosed if stomach lumen visible from oesophagus or gastroesophageal flap valve classified as III/IV during EGD.	1. BO prevalence: 22.5% (SSBO 99.4%; LSBO 0.014%). 2. Risk factors: HH (OR 2.89, *p* < 0.0001), male sex (OR 0.52 for females), H. pylori eradication (OR 1.34), PPI use (OR 1.52), bile reflux (OR 1.18), age ≥ 50 (OR 1.13), NSAID use (OR 1.29). 3. Social drinking reduced BO risk (OR 0.77). 4. Post-H. pylori eradication associated with higher BO risk than H. pylori-negative status.	(1) Limitations: Endoscopic diagnosis without histology; potential H. pylori misclassification. (2) All-Japanese cohort limits generalizability. (3) Large sample but low LSBO prevalence (0.014%).
Kuo et al. (2010) [57]	SIM confirmed via biopsies; Prague C&M criteria for endoscopic classification.	HH: Distance between GOJ and diaphragmatic hiatus > 2 cm.	1. BO prevalence: 1.8% in total study populations and 3.8% in GORD patients. 2. HH (OR = 4.7; *P* = 0.02) and GORD > 5 years (OR = 4.2; *P* = 0.03) were independent risk factors.	Limitations: Self-referred population, excluded asymptomatic BO. Strengths: Standardized biopsy protocol. Confounders: Male predominance in BO group.
Kwon et al. (2020) [58]	N/A	N/A	1. HH associated with higher odds of BO (aOR 10.9) and dysplasia (low-grade aOR 34.5; high-grade aOR 14.7) 2. Odds of undergoing ablation for BO was higher in patients with HH (aOR 4.77).	Database limitations (coding bias, no HH size data). Highlights dysplasia risks
Lee et al. (2010) [59]	CLE ≥ 1 cm with specialized intestinal metaplasia (SIM); SSBO (1–2.9 cm), LSBO (≥ 3 cm)	Distance between diaphragmatic indentation and GOJ > 2 cm	1. BO in 1% of patients 2. HH prevalence 2.73%. 3. HH significantly increased BO risk (OR 6.21, 95% CI 1.78–21.72, *p* = 0.001).	Highlighted low BO prevalence in Asians; GORD symptoms linked to BO risk.
Lord et al. (2009) [21]	Microscopic intestinal metaplasia in a macroscopic columnar-lined oesophagus of any length.	GOJ located ≥ 2 cm proximal to the crural impression at endoscopy.	Patients with BO had significantly higher HH prevalence (84.1%) compared to those with either mild ERD (45.2%) or NERD (53.8%)	Focuses on GORD spectrum; HH size not specified. Surgical outcomes emphasized.
Mathew et al. (2011) [60]	Columnar mucosa with or without specialized intestinal metaplasia (SIM).	Endoscopic diagnosis (no size threshold).	1. BO 16.54%, SIM 8.99% 2. HH associated with SIM (OR 3.95) and BO (OR 3.14)	First Indian study using Prague criteria. HH strongly linked to BO.
Nguyen et al. (2014) [62]	Intestinal metaplasia with goblet cells (biopsy-confirmed)	Presence of HH recorded endoscopically	1. BO prevalence lower in AAs (5% vs. 21.3% in NHWs). 2. HH ≥ 3 cm significant in both (OR 4.12 for AAs; 4.95 for NHWs).	Racial disparities; HH size ≥ 3 cm critical. No dysplasia in AAs.
Öberg et al. (1998) [63]	SIM < 3 cm (SSBO) or ≥ 3 cm (LSBO). Confirmed via biopsies by identification of goblet cells within columnar epithelium	HH: ≥2 cm separation between diaphragmatic crura and GOJ.	1. LSBO had higher prevalence of HH when compared with patients with no mucosal injury and oesophagitis 2. Patients with short-segment Barrett’s oesophagus had elevated esophageal acid and bilirubin exposure, decreased lower esophageal sphincter pressure and length, and a high incidence of hiatal hernia. 3.BO length ↑ with defective LES (*P* < 0.05).	Limitations: Tertiary center bias, no dysplasia data. Strengths: Standardized pH/bilirubin monitoring. Confounders: Male predominance in BO groups.
Ödemiş et al. (2009) [64]	SIM (goblet cells) in esophageal biopsies; endoscopic columnar-lined oesophagus ≥ 3 cm (LSBO) or < 3 cm (SSBO)	HH: Axial length from GOJ to diaphragmatic hiatus ≥ 2 cm (diagnosed via endoscopy)	1. BO prevalence: 1.2% 2. HH linked to higher BO (14% vs. 0.5% in normal GOJ).	Limitations: Small BO sample (*n* = 12); potential underdiagnosis of small HH. Strengths: Standardized endoscopic criteria.
Park et al. (2009) [65]	Columnar epithelium with intestinal metaplasia confirmed by histology	Endoscopic identification (not explicitly defined)	1. BO prevalence: 0.84% (215/25,536). 2. Risk factors: male sex (OR 1.82), NSAID use (OR 2.02), HH (OR 5.66), age ≥ 60 (OR 1.81).	Lower BO prevalence than Western countries; HH a key risk factor.
Peng et al. (2009) [66]	Endoscopic CLE with histologic confirmation (SIM containing goblet cells)	Hiatus hernia defined as absence of muscular ridge at gastric entry, GOJ stays open	1. Prevalence of BO in asymptomatic individuals: 0.5%. 2. HH was a significant risk factor for BO (OR 3.60, 95% CI 1.10-11.77, *p* = 0.034).	Focused on “silent GORD”; included SSBO and LSBO.
Pohl et al. (2013) [67]	Endoscopic appearance + histologic confirmation (intestinal metaplasia)	Endoscopic identification (not explicitly defined)	1. For patients with GORD, HH was associated with BO (OR 2.43) with a significant trend between size and effect (P trend = 0.001) 2. No link between HH and cancer progression in patients with BO. 3. HH size ≥ 3 cm noted but not critical.	Focused on disease progression; HH size categorized but not conclusively linked to BO progression.
Quach et al. (2020) [68]	Columnar epithelium ≥ 1 cm above GOJ	Separation between GOJ and diaphragmatic impression > 2 cm	1. BO prevalence: 2.4%. 2. HH (OR = 7.53, *p* < 0.001) and reflux symptoms (OR = 2.07, *p* = 0.02) were significant factors associated with BO. 3. 2 cases of low-grade dysplasia (no malignancy reported).	Focuses on Asian population; HH > 2 cm as threshold.
Rajendra et al. (2004)[69]	SIM with goblet cells; LSBO (≥ 3 cm), SSBO (< 3 cm).	HH: Distance ≥ 2 cm between gastric folds and diaphragmatic hiatus.	1. BO prevalence: 6.2% (1.6% LSBO, 4.6% SSBO). 2. HH (OR = 5.42; *P* < 0.01) and EE (OR = 2.44, *p* < 0.01) linked to BO. 3. 12.5% LSBO had dysplasia; none in SSBO.	Limitations: Tertiary center bias, potential underdiagnosis of SSBO. Strengths: Multiethnic cohort. Confounders: Socioeconomic factors (higher Indian representation in hospital referrals).
Ringhofer et al. (2008) [70]	Columnar-lined oesophagus (CLE) with intestinal metaplasia (IM) in cardiac mucosa.	HH ≥ 2 cm above diaphragm (endoscopic assessment).	1. All patients had CLE. 2. HH present in 25% 3. HH associated with higher proportion of IM (57.9% vs. 16.9%, *p* = 0.001).	Endoscopy alone insufficient for BO diagnosis; biopsies critical for IM detection.
Ronkainen et al. (2005) [71]	Endoscopic CLE + histologic SIM (goblet cells). LSBO (≥ 2 cm), SSBO (< 2 cm)	Hiatus hernia recorded endoscopically	1. Overall BO prevalence: 1.6% (LSBO 0.5%, SSBO 1.1%). 2. HH was a significant risk factor for LSBO (OR 13.0, 95% CI 1.43–118.0, *p* = 0.01).	Population-based design; included asymptomatic individuals.
Sarr et al. (1985) [72]	Columnar epithelium in lower oesophagus (histopathologic + endoscopic confirmation; excluded hiatus hernia biopsies). Barrett’s mucosa = columnar-lined mucosa with goblet cells	Hiatal hernia recorded via endoscopy (no size criteria)	1.70% of BO patients had HH vs. 48% non-BO (*p* < 0.01). 2.BO + adenocarcinoma: lower HH prevalence (31% vs. 69%, *p* < 0.05). 3. 15% of BO patients had adenocarcinoma.	
Schlottmann et al. (2018) [73]	≥ 1 cm of metaplastic columnar epithelium with intestinal metaplasia (goblet cells)	Measured via barium swallow: no HH, < 3 cm, 3–5 cm, > 5 cm	Larger HH correlates with lower LES pressure (*p* = 0.001), higher acid exposure (DeMeester score up to 146), and increased BO prevalence (50% in HH > 5 cm).	Emphasizes clinical implications for managing large HH; BO risk escalates with HH size.
Sgouros et al. (2007) [74]	Columnar epithelium ≥ 1 cm with intestinal metaplasia (goblet cells confirmed histologically)	Endoscopic measurement: ≥2 cm between diaphragmatic indentation and gastric folds	1. HH prevalence: 21.2% (controls), 60.4% (NERD), 88.2% (BO). 2. HH > 3 cm linked to BO (*P* < 0.05).	HH > 3 cm correlates with severe GORD (BO). No direct dysplasia/cancer analysis.
Sharifi et al. (2014) [75]	Histologic confirmation of intestinal metaplasia	Gastric folds ≥ 3 cm above diaphragmatic pinch	1. BO prevalence: 4.6%. 2. HH strongly associated with BO (OR 4.56, 95% CI 3.3–6.31, *p* < 0.001).	HH and H. pylori infection were key risk factors. No data on HH size or treatment impact.
Toruner et al. (2004) [80]	Intestinal metaplasia in columnar-lined oesophagus.	GOJ ≥ 2 cm proximal to crural impression at endoscopy.	1. BO prevalence: 7.4%. 2. HH present in 65.5% of BO patients vs. 35.2% non-BO (*p* < 0.01).	Symptoms not predictive of BO. HH and antral metaplasia linked to BO.
Wakelin et al. (2003) [81]	Presence of intestinal metaplasia in biopsy specimens from salmon-colored mucosa. Long-segment BO (LSBO): ≥3 cm. Short-segment BO (SSBO): <3 cm.	Hiatal hernia (HH) diagnosed if esophagogastric junction displaced ≥ 1 cm proximal to diaphragmatic hiatus. HH length = distance between diaphragmatic constriction and esophagogastric junction.	1. HH length and BO length: *r* = 0.62 (*p* < 0.01). 2. Acid exposure and BO length: *r* = 0.62 (*p* < 0.01). 3. Regression model: BO length (cm) = 0.79 + (0.68 × HH length) + (0.075 × % time pH < 4) (R² = 0.54, *p* < 0.001). 4. LSBO vs. SSBO: HH length (3.4 cm vs. 1.5 cm; *p* = 0.008); acid exposure (27.8% vs. 9.5%; *p* < 0.01).	(1) Small sample size (*n* = 24); all-male cohort (VA population). (2) HH definition (≥ 1 cm) differs from some studies. (3) Regression model explains 54% of variance; suggests additional factors influence BO length. Important to add duration of oesophageal acid exposure as well
Wang et al. (2008) [82]	Endoscopic suspicion + histologic confirmation of intestinal metaplasia	Endoscopically diagnosed hiatal hernia	1. 48.4% BO confirmed. 2. RFs for BO are long-segment BO that measured >/=3 cm (odds ratio [OR] 4.61 [95% CI, 3.73–5.69]), male sex (OR 1.82 [95% CI, 1.49–2.22]), increasing age (age interval 70–79 years with OR 2.33 compared with age!50 years [95% CI, 1.75–3.10]), the presence of a hiatal hernia (OR 1.46 [95% CI, 1.22–1.84]), and white race (OR 1.90 [95% CI, 1.49–2.22]).	HH and LSBO increased confirmation rates. No dysplasia/malignant data. Biopsy sampling limitations noted.
Weston et al. (1999) [85]	Endoscopic and histologic confirmation of columnar epithelium with goblet cells.	Presence of hiatal hernia diagnosed endoscopically.	1. 7.1% of patients achieved complete regression. 2. Stepwise logistic regression analysis revealed that complete regression was significantly and independently associated with absence of a hiatal hernia (*p* = 0.025).	Focused on regression predictors; all participants were male veterans.
Xiong et al. (2010) [87]	Histologic confirmation based on the presence of a goblet cell in intestinal metaplasia. SSBO < 3 cm above GOJ	Presence of hiatal hernia diagnosed endoscopically and confirmed by barium swallow	1. Prevalence of BO = 1% (21/2022) 2. Prevalence of BO in patients with HH was 3.4%(1/29) vs. 1% (20/1993) (*p* = 0.197) 3. Age (OR 1.03) and reflux oesophagitis (OR 4.44) were factors associated with BO	A similar result was found in the prevalence of hiatus hernia between BO and non-BO patients. This may be because of the low number of BO cases. The low prevalence of heartburn and hiatus hernia in Chinese patients might also a possible reason.
Yilmaz et al. (2006) [88]	Histologic confirmation of intestinal metaplasia with goblet cells	Hiatal hernia recorded via endoscopy (no size criteria)	1. BO prevalence: 0.4% (histologic). 2. HH in 4.8% of BO cases vs. 11.2% in suspected BO (p = NS).	Lower HH prevalence in confirmed BO. No data on malignant progression, HH size, or treatment implications.

The size of HH appears to be an important factor in BO development. HHs larger than 2 cm were associated with more than a five-fold increased risk of developing BO [51], and this risk remained significant with HH ≥ 3 cm in both African Americans (OR = 4.12) and non-Hispanic Whites (OR = 4.95), as reported by Nguyen et al [62]. Additionally, Schlottmann et al. reported a 50% prevalence of BO when the HH exceeded 5 cm in size [73]. Larger HHs were also associated with longer BO segments, as shown in several studies [35, 39, 46, 74, 81].

HH appears to be a particularly significant risk factor for long-segment BO[55]. One study reported an almost eighteen-fold increased risk of long-segment BO when HH size exceeded 4 cm [40]. The presence of HH was also far more common in patients with long-segment BO [44, 48, 49, 63], with one study reporting HH in 100% of long-segment BO cases compared to 48.6% in short-segment BO [48].

Beyond its association with BO onset, HH is also linked to dysplasia on index diagnosis [32, 58]. Kwon et al. found significantly higher odds of both low-grade dysplasia (LGD; adjusted OR 34.5) and high-grade dysplasia (HGD; adjusted OR 14.7) in patients with HH [58]. Furthermore, large HH size in male patients was independently associated with HGD (OR 12.18) [32]. These findings underscore that both the presence and size of HH are critical determinants in the development of BO and dysplasia.

### Risk of dysplastic and malignant progression in BO

A total of nine studies investigated the association between HH and dysplastic progression of BO (Table 3). The findings were inconsistent, with most studies [29, 42, 61, 76, 77, 79] reporting no significant association between the presence or size of HH and progression to high-grade dysplasia (HGD), even after adjusting for potential confounders. However, two studies identified HH size as a possible risk factor. Avidan et al. reported that HH ≥ 2 cm was associated with increased odds of HGD (OR 1.2 per cm increase, 95% CI 2.13–2.89)[30]. Similarly, Weston et al. found that large HHs (≥ 6 cm) were significantly associated with higher risk of HGD (OR 4.51) [84].

**Table 3:** Association of hiatus hernia with dysplastic progression and adenocarcinoma of barrett’s oesophagus

Association of Hiatus Hernia with Dysplastic Progression of Barrett’s Oesophagus				
Authors	Definition of BO	Definition of HH	Important results	Notes
Solanky et al. (2019 )[77]	Intestinal metaplasia confirmed by expert GI pathologists	Hiatal hernia recorded via endoscopy (no size criteria)	1. Hiatal hernia length: Mean 4.00 cm in progressors vs. 3.79 cm in nonprogressors (*P* = 0.51). Not significant in analysis. 2. Hiatal hernia size not significant as predictors of progression to HGD/EAC on univariate analysis (HR 1.02 95%CI 0.82–1.23)	HH length was not an independent predictor of progression. Focused on BO length, nodularity, and baseline LGD.
Sikkema et al. (2011) [76]	Endoscopic BO ≥ 2 cm + histologic intestinal metaplasia	Hiatal hernia presence/size recorded; mean length: 3.6 cm (range 1.0–14.0 cm)	1. HH prevalence: 87% (617/713). 2. No association between HH length and progression (RR 0.9, 95% CI 0.7–1.2; *P* = 0.821). 3. Key predictors: LGD, BO duration ≥ 10 years, longer BO length, esophagitis.	1. HH common but not a progression predictor. 2. No HH size-specific risk analysis. 3. HH treatment impact not addressed.
Avidan et al. (2002) [35]	Specialized columnar epithelium with intestinal metaplasia (endoscopic biopsy)	Hiatal hernia ≥ 2 cm above diaphragmatic hiatus	1.HGD/CA vs. controls w/o GORD: hiatal hernia size (cm) OR 2.48, 95% CI 2.13–2.89, *p* < 0.05 2. HGD/CA vs. controls w/ BO: hiatal hernia size (per cm length) OR 1.2, 95%CI 1.04–1.39, *p* = 0.013	Multifactorial risk (HH size, BO length, acid reflux). Male gender and white ethnicity also notable factors.
Alnasser et al. (2019) [29]	Columnar-lined mucosa with intestinal metaplasia (endoscopic and pathologic confirmation)	Hiatal hernia recorded via endoscopy (no size criteria)	Presence of hiatal hernia was not a significant factor for progression from nondysplastic BO to high-grade dysplasia or oesophageal adenocarcinoma	HH presence not significant. Focus on BO length, age, and mucosal irregularities. Limited data on HH specifics.
Pohl et al. (2013) [67]	Endoscopic appearance + histologic confirmation (intestinal metaplasia)	Hiatal hernia recorded via endoscopy (no size criteria)	HH was not a risk factor for the development of cancer/HGD in Barrett’s patient	Focused on disease progression; HH size categorized but not conclusively linked to BO progression.
Thiruvengadam et al. (2020) [79]	Salmon-colored mucosa ≥ 1 cm with intestinal metaplasia.	Presence/size (small, medium, large) recorded during endoscopy.	HH presence not significant in univariate analysis (*p* = 0.65).	Endoscopic factors (HH, segment length) were non-significant after adjustment. Focus shifted to histologic factors (e.g., dysplasia).
Mohamed Hussein et al. (2021) [61]	Confirmed by expert histopathologists (specialized intestinal metaplasia)	Hiatus hernia (HH) presence; size categorized as < 3 cm (small) or > 3 cm (large)	HH presence (*P* = 0.83) and size (*P* = 0.60) not associated with progression	HH analyzed but not significant; size categories mentioned but no explicit definition
Coleman et al. (2014) [42]	Columnar-lined oesophagus with specialized intestinal metaplasia	Hiatal hernia recorded via endoscopy (no size criteria)	HH presence not significant for dysplastic or neoplastic progression (HR 0.96, 95% CI 0.66–1.39, *P* = 0.82) in adjusted models	HH analyzed but not significant; no size stratification
Weston et al. (2004) [84]	Histologically confirmed intestinal metaplasia in distal oesophagus	Hiatal hernia recorded via endoscopy (no size criteria)	Larger HH (≥ 6 cm) associated with higher risk of index HGD/cancer (OR 4.51) and progression (HR 0.42)	Emphasizes HH size as critical for risk stratification; recommends intensified surveillance for large HH.
**Association of Hiatus Hernia and Malignant Progression of Barrett’s Oesophagus**				
**Authors**	**Definition of BO**	**Definition of HH**	**Important results**	**Notes**
Bani-Hani et al. (2005) [37]	Columnar-lined epithelium ≥ 3 cm or specialized columnar epithelium (SCE) in biopsies	Endoscopically identified hiatal hernia	HH was not a significant risk factor for the development of oesophageal adenocarcinoma on univariate analysis (*P* = 0.383).	HH prevalence: 50% in cancer group vs. 56.8% in non-cancer group (NS).
Pohl et al. (2013) [67]	Endoscopic appearance + histologic confirmation (intestinal metaplasia)	Endoscopic identification (not explicitly defined)	HH was not a risk factor for the development of cancer/HGD in Barrett’s patient	Focused on disease progression; HH size categorized but not conclusively linked to BO progression.
Sikkema et al. (2011) [76]	Endoscopic BO ≥ 2 cm + histologic intestinal metaplasia	Hiatal hernia presence/size recorded; mean length: 3.6 cm (range 1.0–14.0 cm)	1. HH prevalence: 87% (617/713). 2. No association between HH length and progression (RR 0.9, 95% CI 0.7–1.2; *P* = 0.821). 3. Key predictors: LGD, BO duration ≥ 10 years, longer BO length, esophagitis.	1. HH common but not a progression predictor. 2. No HH size-specific risk analysis. 3. HH treatment impact not addressed.
Krishnamoorthi et al. (2016) [54]	Defined via GPRD diagnostic codes (no histology requirement).	Presence coded using GPRD diagnostic codes.	1. Incidence of EC: 2.23/1000 person-years. 2. Risk factors: male gender (HR 2.79), age (HR 1.04/year), overweight (HR 1.63). 3. Protective factors: PPI (HR 0.43) and statin use (HR 0.61). 4. HH not significantly associated (HR 0.99, *p* = 0.958).	Limitations: No histology data; HH size not assessed.
Coleman et al. (2014) [42]	Columnar-lined oesophagus with specialized intestinal metaplasia	Hiatal hernia recorded via endoscopy (no size criteria)	HH presence not significant for neoplastic progression (HR 0.96, 95% CI 0.66–1.39, *P* = 0.82) in adjusted models	HH analyzed but not significant; no size stratification
Iwaya et al. (2019) [50]	Prague criteria: LSBO (C ≥ 3 cm), SSBO (C < 3 cm); intestinal metaplasia with goblet cells confirmed.	HH presence recorded via endoscopy (no size criteria).	1.LSBO EAC: 84% HH vs. 73% SSBO (*p* = 0.02). 2. Patients with HH has an increased likelihood of having EAC develop from LSBO than SSBO (OR 1.87, 95% CI 1.02–3.45, *p* = 0.04)	
Weston et al. (2004) [84]	Histologically confirmed intestinal metaplasia in distal oesophagus	Hiatal hernia recorded via endoscopy (no size criteria)	Larger HH (≥ 6 cm) associated with higher risk of index HGD/cancer (OR 4.51) and progression (HR 0.42)	Emphasizes HH size as critical for risk stratification; recommends intensified surveillance for large HH.
Wu et al. (2003) [86]	Not explicitly defined	Physician-diagnosed hiatal hernia	HH increased risk of oesophageal adenocarcinoma (OR 5.85, 95%CI 3.18–10.75 for HH without reflux conditions; OR 8.11, 95%CI 4.75–18.87 with both).	Focuses on adenocarcinoma
Sarr et al. (1985) [72]	Columnar epithelium in lower oesophagus (histopathologic + endoscopic confirmation; excluded hiatus hernia biopsies). Barrett’s mucosa = columnar-lined mucosa with goblet cells	Hiatal hernia recorded via endoscopy (no size criteria)	1.70% of BO patients had HH vs. 48% non-BO (*p* < 0.01). 2.BO + adenocarcinoma: lower HH prevalence (31% vs. 69%, *p* < 0.05). 3. 15% of BO patients had adenocarcinoma.	

Likewise, nine studies examined the relationship between HH and malignant progression of BO (Table 3), and similar inconsistencies were observed. While several studies found no significant link between HH and the development of oesophageal adenocarcinoma (OAC) [37, 42, 54, 67, 76], others suggested that the risk may depend on specific clinical contexts. For instance, Wu et al. reported a markedly increased risk of OAC when HH was present in combination with reflux symptoms (OR 8.11, 95% CI 4.75–18.87)[76]. Additionally, larger hernias (≥ 6 cm) were independently associated with elevated OAC risk (OR 4.51)[84]. Another study found that HH conferred an increased risk of OAC specifically in patients with long-segment BO (OR 1.87, 95% CI 1.02–3.45)[50].

### Impact of HH on BO treatment outcomes

The impact of HH on the treatment outcomes of BO appears to be variable (Table 4). Two studies[27, 55] reported that larger HH size was associated with persistent IM following RFA. Additionally, patients with larger hernias who responded to ablation required more RFA sessions and were more likely to experience non-healing (median HH size: 7 cm)[53]. In contrast, three other studies[7, 53, 83] found no significant association between HH presence or size and poor RFA treatment outcomes.

**Table 4:** Impact of hiatus hernia on the treatment of barrett’s oesophagus

Authors	Definition of BO	Definition of HH	Important results	Notes
Akiyama et al. (2012) [27]	Prague C & M criteria + biopsy-confirmed metaplasia	Endoscopically measured (cm)	1. Patients with smaller HH were more likely to have CE (0.5 cm vs. 2.0 cm in CE vs. non-CE, *p* = 0.045) 2. HH size (OR 0.45, 95%CI 0.23–0.86, *p* = 0.02) and acid control independent predictors of CE	Recommends HH repair and pH optimization for better RFA outcomes; HH not correlated with acid exposure The underlying mechanism for this is still not clear, but it was independent of acid since there was no significant correlation between hiatal hernia size and the amount of EAE in our patients. One hypothesis could be that the widening of the distal oesophagus into a hiatal hernia may make it difficult to bring the electrode of the HALO360 catheter or the HALO90 pad into good circumferential/ focal contact with the mucosa at the GOJ, resulting in insufficient ablation of BO at this level. Since, according to some studies, laparoscopic fundoplication is associated with better ablation outcomes when combined with RFA [30, 34] and may correct both the abnormal EAE and the underlying hiatal hernia, it would be an attractive option in such patients.
Korst et al. (2011) [53]	Columnar segment > 3 cm or < 3 cm with intestinal metaplasia	Sized endoscopically (distance from incisors to gastric folds minus crural pinch)	1. Larger HH (median 7 cm) experienced nonhealing following initial ablation 2. Patients who were successfully ablated but had larger HH and longer segments required more RFA sessions (*p* = 0.003 and *p* = 0.007) 3. Size of HH not linked to success or failure of RFA (*p* = 0.38)	HH size ≥ 7 cm associated with treatment failure; recommends HH repair for large HH. Non-healing represents a major cause of technical failure
Krishnan et al. (2012) [55]	Specialized columnar mucosa with goblet cells (intestinal metaplasia)	Presence and size measured endoscopically; median HH size: 2.3–3.0 cm	Larger HH (3.0 cm in ICR vs. 2.3 cm in CR, *p* < 0.01) linked to persistent IM post-RFA.	Uncontrolled reflux (weakly acidic) despite PPIs increases IM persistence. Recommends reflux optimization.
Soroush et al. (2019) [78]	BO with intestinal metaplasia (baseline histology: HGD/IMCA in 74.2%)	Hiatal hernia size (median 2 cm, IQR 1–3 cm)	1. Achieving CE-IM within 18 months: larger hiatal hernias were independently associated with higher risks of both dysplasia/cancer recurrence (adjusted HR = 1.23 per cm) 2. CE-IM: larger HH size associated with dysplasia or EAC recurrence (adjusted HR 1.37 per cm; 95% CI 1.08–1.72) and increased risk of IM recurrence (adjusted HR 1.15 per cm; 95% CI 1.01–1.31)	Suggests antireflux surgery may improve outcomes in larger HH.
Tsoi et al. (2020) [7]	BO with dysplasia treated with RFA	Presence of HH assessed endoscopically	Presence of HH and size of HH were not predictors for poor response to RFA	HH size not significant; aggressive reflux control recommended.
Weiss et al. (2023) [83]	Dysplastic BO (Prague classification: C and M values)	Endoscopic determination of HH (no size criteria)	1. Absence of HH associated with RFA failure in univariate analysis (*p* < 0.05). 2. HH presence in 80% of patients. 3. No significance in multivariate analysis.	Confounding factors (e.g., PPI adherence) may influence HH association. These unexpected finding could result from a reporting bias, or from a confusion bias. Indeed, patients with hiatal hernia tend to report more symptoms than others, and therefore could be more likely to take their PPI treatments, leading to improved squamous regeneration in the hiatal hernia group.

## Discussion

BO is a premalignant condition that can progress to OAC through a well-recognised stepwise sequence[6]. Early identification of individuals at risk for BO offers an opportunity to reduce the incidence of OAC through targeted risk modification. Several national and international bodies [19–22] recommend screening for BO in individuals with multiple risk factors and ongoing surveillance once a diagnosis is established. Recognised risk factors include chronic gastro-oesophageal reflux disease, male sex, age over 50 years, smoking, obesity, and a family history of BO or OAC [15–18, 89]. However, HH is not currently considered a major risk factor in these guidelines. Therefore, this systematic scoping review aims to evaluate the clinical significance of HH in the context of BO, focusing on its association with disease development, progression, and treatment outcomes. By synthesising current evidence, we seek to support surgical decision-making and explore whether HH, even in asymptomatic patients, should be considered for operative management to potentially reduce the risk of BO progression. To our knowledge, this is the first scoping review to address these questions.

Current evidence underscores HH as a significant risk factor for the development of BO. This association aligns with the established pathogenesis of BO, which involves chronic exposure of the oesophageal mucosa to gastric and bile acids resulting from disruption of the anti-reflux barrier [90]. HH contributes to reflux through several key mechanisms: it alters the anatomy of the GOJ by distorting the angle of His, weakening the gastro-oesophageal flap valve [91], and reducing lower oesophageal sphincter (LOS) competence via a widened diaphragmatic hiatus [92]. Additionally, the hernia sac can trap refluxate, impairing oesophageal acid clearance [93]. Hernia size further exacerbates reflux and has been associated with increased length of BO segment [44, 48, 49, 63] and a higher risk of dysplasia [32, 58]. Larger hernias are particularly associated with more severe reflux and oesophagitis [94] driven by lower LOS pressure [95], higher acid exposure [73] and the absence of an intra-abdominal oesophageal segment [96]. While no universal hernia size threshold exists, axial hernia lengths of ≥ 2 cm [51] are associated with increased BO risk, and hernias ≥ 4 cm may correlate with longer BO segments [40] and higher grades of dysplasia [32].

The association between HH and the dysplastic or malignant progression of BO, meanwhile, remains inconsistent. While most studies have not demonstrated a significant link, two have identified HH size (≥ 6 cm) as a risk factor for HGD [84] and OAC [50, 84]. These discrepancies likely arise from heterogeneity in study designs, inconsistent definitions of BO and HH, and inadequate adjustment for confounders such as GORD severity, proton pump inhibitor (PPI) use, obesity, and BO segment length. Additionally, the underreporting of HH in endoscopy documentation may contribute to this variability. A synergistic interaction between HH and reflux symptoms was observed by Wu et. al.[86], resulting in an eight-fold increase in OAC risk—an observation not evaluated in other studies. This suggests that it may be the combined effect of HH and reflux symptoms, rather than HH alone, that drives carcinogenesis.

Management of BO, with or without dysplasia, typically involves PPI therapy and/or endoscopic eradication therapy[1]. The presence of a HH may compromise the effectiveness of endoscopic treatment through both mechanical and reflux-related mechanisms. Anatomically, HH can cause widening and tortuosity of the distal oesophagus at the GOJ in HH. This makes the treatment challenging as the ablation catheter may not have good mucosal apposition at the targeted area [7, 27]. This is more pronounced in larger hernias where axial movement of the GOJ is increased [55]. A HH measuring 2 cm or more is reportedly significant enough to impair complete eradication of BO following RFA treatment [27]. The end results are incomplete ablation of dysplastic tissue and prolonged or repeated RFA sessions [53].

HH serves as a marker for severe reflux pathophysiology, contributing to increased exposure to both acid and bile [97]. Patients who have successful ablation of BO can also be vulnerable to recurrence due to increased permeability of neosquamous oesophageal epithelium to refluxate [98]. PPI therapy use is aimed to prevent continuous damage of oesophageal mucosa and achieve effective symptom control. However, up to 20% of patients on high-dose PPI continue to exhibit abnormal oesophageal acid exposure [55]. PPI treatment also does not suppress weakly acidic and alkaline bile salt reflux [99, 100]. Impaired clearance of refluxate further perpetuates mucosal injury and reduces the likelihood of complete eradication of IM [78]. These findings highlight the dual challenge posed by HH, which are mechanical interference with endoscopic therapy and its role in sustaining a reflux-prone microenvironment. Hence, optimised management may require tailored strategies such as surgical correction to improve therapeutic outcomes.

Surgery remains the most definitive, proven treatment for HH and GORD. When compared to PPI treatment, laparoscopic fundoplication is more superior in maintaining complete endoscopic resolution of BO following ablation therapy [101]. This suggests a potential synergistic role in patients with HH by restoring the esophagogastric anatomy and mitigating acidic and alkaline reflux, which cannot be adequately addressed by PPI treatment. Current standard practice generally reserves HH repair for symptomatic sliding or paraesophageal hernias, while elective repair of asymptomatic paraesophageal HH is decided on an individual basis, considering age, comorbidities, and surgical risk [102]. Our findings suggest this approach may require individualisation in the context of BO. HH, particularly when ≥ 4 cm, is a significant risk factor for BO development with larger hernias (> 6 cm) and worsening symptoms posing potentially increased malignant risk. For patients with HH and established BO, prophylactic surgical repair should be considered regardless of symptoms to improve clinical outcomes. This underscores the need for better risk stratification that incorporates hernia characteristics alongside patient factors. In cases of large, symptomatic hernias with extensive dysplasia, some authors have proposed oesophagectomy as the definitive treatment [53].

This review has several limitations. First, heterogeneity in the diagnostic criteria for BO limits the comparability of findings across studies. Many rely on traditional goblet cell–based definitions, which may obscure associations between HH and BO. Up to 73% of dysplastic lesions in BO may arise via a non-goblet cell (“gastric” or “foveolar”) pathway [103], characterised by mucinous cytoplasm, basally located nuclei, and expression of gastric markers (MUC5AC, MUC6) [104]. These lesions often lack intestinal markers (CDX2, MUC2) [105] and are frequently misclassified as reactive due to their subtle morphology [106]. Despite their low-grade appearance, foveolar-type dysplasia demonstrates aggressive biological behaviour and can progress to carcinoma even in the absence of goblet cells; nearly half of such cases lack goblet cells in the surrounding mucosa [105]. Consequently, existing studies may underestimate HH’s role in BO progression by focusing solely on intestinal-type dysplasia and excluding foveolar-type lesions. Given that the diagnosis of foveolar dysplasia requires immunohistochemical analysis—rarely employed in routine pathology—HH’s potential contribution to neoplastic progression may remain unrecognised. The absence of a demonstrated association may therefore reflect limitations in current diagnostic practices rather than a true lack of risk, underscoring the need for revised diagnostic frameworks that encompass all dysplastic pathways. Second, the type and size of HH were often not specified, which may influence both reflux severity and treatment outcomes. Third, although numerous studies identified an association between HH and BO, this relationship frequently lost significance after adjustment for key confounders, particularly GORD and obesity. This suggests that the apparent risk may be largely mediated through, or shared with, these coexisting conditions. The role of HH as an independent driver of carcinogenesis remains unproven, limiting the strength of the conclusions. Finally, variability in the study populations contributes to selection bias, and within the limits of process involved in scoping review, the recommendations are based on available low-quality evidence.

## Conclusions

HH is an under-recognised yet clinically significant contributor to the pathogenesis of BO. Evidence supports a strong association between HH, particularly large hernias, and BO development. There could be a trend towards dysplastic and malignant progression of BO in HH. Surgical repair may have a potential role in selected asymptomatic patients, especially those with hernias ≥ 4 cm and established BO, to optimise clinical outcomes. Further evidence is needed before firm recommendations can be made. Future studies should adopt standardised definitions and diagnostic criteria for both BO and HH to better guide clinical decision-making. 

## Supplementary Information

Below is the link to the electronic supplementary material.

Supplementary Material 1 (DOCX 17.3 KB)

## Data Availability

The data supporting the findings of this study are available within the article and its supplementary materials. To request data from this study, please contact Dr Lee Shyang Kyang (lees.kyang@gmail.com).

## Abbreviations

HH: Hiatus hernia
BO: Barrett’s oesophagus
EMR: Endoscopic mucosal resection
RFA: Radiofrequency ablation
CLE: Columnar-lined epithelium
IM: Intestinal metaplasia
GOJ: Gastro-oesophageal junction
OR: Odds ratio
LGD: Low-grade dysplasia
HGD: High-grade dysplasia
OAC: Oesophageal adenocarcinoma
LOS: Lower oesophageal sphincter
GORD: Gastro-oesophageal reflux disease
PPI: Proton-pump inhibitor
