# Supplementary material for: Clinical significance of hiatus hernia on Barrett’s oesophagus: a scoping review
Source: Langenbecks Arch Surg. 2026 Feb 19;411(1):97. doi: 10.1007/s00423-026-03981-z (PMC12975827; doi:10.1007/s00423-026-03981-z)
Supplement: Supplementary file 1 — Supplementary Material 1 (DOCX 17.3 KB) [file 423_2026_3981_MOESM1_ESM.docx]

**SEARCH STRATEGY**

**PUBMED**

| Search words | Results |
| --- | --- |
| 1. “hiatus hernia” OR “hiatal hernia*” OR “paraesophageal hiatal hernia*” OR “paraesophageal hernia*” OR “sliding hiatal hernia*” OR “sliding esophageal hernia*” OR “esophageal hernia*” | 10,798 |
| 1. “barret* oesophagus” OR “barret* esophagus” OR “barrett* syndrome” OR “barrett metaplasia*” OR “barrett epithelium” | 11,973 |
| 1. “hernia, hiatal”[Mesh] | 7,118 |
| 1. “barrett esophagus”[Mesh] | 8,977 |
| 1. (#1 OR #4) AND (#2 OR #5) | 707 |

**MEDLINE**

| Search words | Results |
| --- | --- |
| 1. “hiatus hernia” OR “hiatal hernia*” OR “paraesophageal hiatal hernia*” OR “paraesophageal hernia*” OR “sliding hiatal hernia*” OR “sliding esophageal hernia*” OR “esophageal hernia*” | 4,340 |
| 1. “barret* oesophagus” OR “barret* esophagus” OR “barrett* syndrome” OR “barrett metaplasia*” OR “barrett epithelium” | 9,599 |
| 1. Hernia, hiatal/ | 3,503 |
| 1. Barrett esophagus/ | 8,109 |
| 1. 1 or 3 | 5,099 |
| 1. 2 or 4 | 9,509 |
| 1. 5 and 6 | 559 |

**EMBASE**

| Search words | Results |
| --- | --- |
| 1. “hiatus hernia” OR “hiatal hernia*” OR “paraesophageal hiatal hernia*” OR “paraesophageal hernia*” OR “sliding hiatal hernia*” OR “sliding esophageal hernia*” OR “esophageal hernia*” | 21,984 |
| 1. “barret* oesophagus” OR “barret* esophagus” OR “barrett* syndrome” OR “barrett metaplasia*” OR “barrett epithelium” | 22,849 |
| 1. Hiatus hernia/ | 18,843 |
| 1. Exp Barrett esophagus/ | 20,615 |
| 1. 1 or 3 | 21,984 |
| 1. 2 or 4 | 22,863 |
| 1. 5 and 6 | 2,078 |

**SCOPUS**

| Search words | Results |
| --- | --- |
| “hiatus hernia” OR “hiatal hernia*” OR “paraesophageal hiatal hernia*” OR “paraesophageal hernia*” OR “sliding hiatal hernia*” OR “sliding esophageal hernia*” OR “esophageal hernia*”  **AND**  “barret* oesophagus” OR “barret* esophagus” OR “barrett* syndrome” OR “barrett metaplasia*” OR “barrett epithelium” | 4,743 |
